# Assessing Wellbeing in People Living with Dementia Using Reminiscence Music with a Mobile App (Memory Tracks): A Mixed Methods Cohort Study

**DOI:** 10.1155/2019/8924273

**Published:** 2019-09-05

**Authors:** Stuart Cunningham, Mark Brill, J. Harry Whalley, Rebecca Read, Gordon Anderson, Sarah Edwards, Richard Picking

**Affiliations:** ^1^Centre for Advanced Computational Science, Manchester Metropolitan University, Manchester M1 5GD, UK; ^2^Faculty of Arts, Science and Technology, Wrexham Glyndwr University, Wrexham LL11 2AW, UK; ^3^School of Communication Design, University for the Creative Arts, Farnham GU9 7DS, UK; ^4^School of Film, Media and Performing Arts, University for the Creative Arts, Farnham GU9 7DS, UK; ^5^Memory Tracks Ltd., London SE3 0DG, UK; ^6^Pendine Park Care Organisation, Wrexham LL11 4YE, UK

## Abstract

The number of people living with dementia is growing, leading to increasing pressure upon care providers. The mechanisms to reduce symptoms of dementia can take many forms and have the aim of improving the wellbeing and quality of life of the person living with dementia and those who care for them. Besides the person who has dementia, the condition has a profound impact upon their loved ones and carers. One therapeutic approach is the use of music, an area recognised as having potential benefit, but requiring further research. The present paper reports upon a mixed methods cohort study that examines the use of a musical mobile app as a way to promote song-task association in people living with dementia. The study took place in care home environments in the UK. A total of fourteen participants (*N* = 14) were recruited. Quantitative measurements were taken on a daily basis prior to, and during, use of the mobile app over several weeks. Metrics came from the complete Self-Assessment Manikin scale (arousal, valence, and dominance), and a subset of three from the Quality of Life in Alzheimer's Disease questionnaire (physical health, memory, and life as a whole). Subsequently, semistructured interviews were conducted with staff at the care home to assess the impact of the app upon their role and the residents they care for. No significant differences were found in the combined quantitative measures for the ten (*n* = 10) sets of responses sufficient to be analysed. However, the qualitative results suggest that use of the mobile app produced positive changes in terms of behaviour, ability, and routine in the life of residents living with dementia. These findings contribute to the growing body of evidence-based research in the field of musical therapies for reducing symptoms of dementia and highlight elements where further study is warranted.

## 1. Introduction

Dementia incidence in the United Kingdom (UK) in people aged over 65 years is over 7%, and the total number of people with dementia in the UK is forecast to reach in excess of 1 million by 2025 and over 2 million by the year 2050, with Alzheimer's disease currently being the most common subtype followed by vascular dementia [1]. On the global scale, dementia is estimated to affect over 75 million people by 2030 and more than 135 million by 2050 [2]. Identifying mechanisms to help manage the symptoms and support those living with dementia is an increasing public health priority.

In addition to the wellbeing of the person living with dementia, there is also recognition of the strains put upon their care givers. For example, work has been conducted that examines the benefits of technological interventions specifically to support care givers, which has indicated that relationships between the carer and patient can be improved as a result [3].

### 1.1. Music and Care

Established research has examined music as a recall trigger for autobiographical memories in patients with Alzheimer's [4], showing that there is potential to use music as a trigger when rebuilding memory. Furthermore, Cuddy et al. [5] supported these findings by studying the impact of melodies and lyrics from music on the recall of certain words in Alzheimer's and dementia patients. Both of these studies show how music can be used in memory recall and as a stimulus.

We hypothesise that there may be an opportunity to use the benefits of music as a trigger to support people living with dementia to engage with activities of daily living. The question that led to the Memory Tracks platform being developed, and the subsequent research described in this article, was posed by Anderson [6]. She suggested that reminiscence music could have a beneficial influence on those living with dementia to undertake daily tasks. The concept of *song-task association* proposed that memory could be supported by associating short pieces of music to specific daily activities for people living with dementia. Anderson suggested that the most memorable musical pieces were those heard in the earlier child development, between four and twelve years of age [6]. These pieces could become triggers associated to tasks for someone living with dementia, especially to those activities that present the greatest challenges such as eating, taking medication, or personal care. Thus, song-task association could demonstrate benefits in reducing the confusion associated with many forms of dementia and, as a result, to reduce levels of agitation or distress.

Other researchers have posited similar ideas and conducted investigations on this topic. For instance, Craig [7] provided a range of compelling arguments, mechanisms, and evaluation methods for the use of music in the field of occupational therapy, citing potential benefits to the physical, mental, and social wellbeing of the individual. More specific to the field of dementia, Raglio et al. [8] examined the impact of music therapy delivered in a traditional form of group sessions. This intervention was shown to reduce the severity of behavioural and psychiatric symptoms in those participants living with moderate to severe dementia. The present study is distinct from these other works insomuch as it was designed to examine the use of music therapy at the point of care and to test the mechanism of song-task association. In doing so, we situated our research in real-life scenarios to enable user-centred evaluation and impact measurement in daily care situations, including feedback from those with caring responsibilities. This is commensurate with a longer-term aim of Memory Tracks, where it is hoped that the platform and research will expand into supporting those living with dementia independently and outside of a care home environment.

A recent study on the amygdala highlights that new memories and association in healthy older adults can be difficult to form if an individual has a cognitive impairment [9]. In order for this association to become an automatic process, sensory experiences must be transferred from working memory or short-term memory (STM) to long-term memory (LTM) [10, 11] and be able to be recalled to working memory when needed again. For our hypothesis to be supported, an association needs to be made in adults living with Alzheimer's and dementia between a specific song and a task in their daily routine. In the process of building this association the use of explicit and implicit memory is required.

Explicit memory is a large part of language recall, and implicit memory is regularly used for word identification. Jäncke [12] explained the importance of these memory facets when building a lasting association; however, it is acknowledged that explicit and implicit memory abilities are diminished by Alzheimer's or other dementia. This could mean that building of the association, which is the primary aim of the present study, will not happen as easily or as rapidly as it would in healthy older adults [13].

The emotional behaviours associated with Alzheimer's and dementia are patterns that occur when someone living with the condition is overwhelmed with emotion [14]. These emotions include confusion, fear, panic, and anxiety, which manifest in behaviours such as lashing out, physical shaking, inability to move, crying, shouting, and screaming. These actions would be classed as emotional behaviours that this research project aims to subdue.

Zhou and Sully [15] explained how the impact of cognitive disease can change the control an individual has over their amygdala and frontal lobe, therefore causing a change in the behaviours that are controlled by the emotional rational part of the frontal lobe. This explains why emotional behaviours are heightened to a point where they impact daily tasks for people living with dementia [16]. Moretti et al. [17] reported research on frontal lobe activation and its influence on emotional behaviours. They suggested there were changes in an individual's behaviours, before and after the decrease in cognitive function has begun, which were gradually manipulated to become an irrational response to task, people, and activities. However, Jäncke [12] suggested it was a natural reaction to fear and panic as the fight or flight instinct would be prominent in Alzheimer's or dementia subjects when they were in an uncomfortable situation. Research undertaken by Wall and Duffy [18] found that behaviours of cognitive defect supported this, yet they also claimed that those reactions were only seen as emotional behaviours (and not fight or flight instinct) due to the magnitude of care which the person living with dementia was receiving.

### 1.2. Reminiscence Music

Previous studies have shown benefits from the use of reminiscence music, such as a reduction in depressive symptoms in those living with dementia [19]. The use of music in the treatment and care of those with physical and cognitive illness is not a new concept and has been practiced in recognisable forms since the nineteenth century [20]. A recent meta-analysis found that musical interventions are shown to have positive effects on quality of life for people living with dementia, although research in the field can be restricted because of variation in quality of the research methodology [21]. This is corroborated by a systematic review from the prestigious Cochrane Collaboration [22], which expresses limited confidence in the ability of music to have an impact upon the wellbeing of people living with dementia. This finding too is due, in part, to the variation in research quality. However, the authors articulate that additional methodically sound research in this field is important to gaining greater understanding of the true benefits of music as an intervention for those with dementia.

Hamon et al. [23] explained why music from an individual's childhood tends to be the music that can recall memories most frequently. This lends its support to the use of music from childhood in the Memory Tracks application. We hypothesise that the use of music from early years will have a strong emotional connection for the participants of the present study. It could not only strengthen the ability to form or recall a new association but also give strength to the memories and associations they already have. That, in turn, may reduce panic or fear in the participants. Koelsch et al. [24] disagreed and argued that music the individual has chosen themselves in the later years of their life may have a larger impact on their recall of certain memories. Although Koelsch et al.'s theory may have some validity, an absence of established evidence means it is difficult to validate the claim. Furthermore, Sung et al. [25] suggested that the preference for choosing music later in life may be the case for a conscious mind, with no cognitive impairment, as the person can control which emotions and memories they recall. Other research [26, 27] on music and personality would agree, although these examples of established research were conducted on healthy older adults, within the same age range as those in this study, but not on older adults living with cognitive impairments such as dementia. Often, when an individual begins to experience the onset of the later stages of dementia, they will regress to memories of childhood and their parents that music from their childhood could help to recall. Therefore, it will prompt the individual to make any emotional recall that is already present.

Autobiographical memory which is memories that relate to specific events, emotions, and other character traits that relate to a sense of identity [28] increases with age [29], with “…*access to semantic or other non-episodic information [being] preserved or facilitated.*” The development of the sense of “self” [30] is built upon by the work of Nelson [31] who theorises six levels of understanding from birth to age seven: physical, social, cognitive, representational, narrative, and cultural. Through the repetition of hearing, playing, or singing music during childhood and youth, music becomes intertwined with the sense of identity and autobiography. The “memory bump” [32] and a more recent systematic review by Munawar et al. [33] reveal that childhood memories particularly persist into old age, and musical memories are no different.

Cuddy et al. [34] demonstrated that music-evoked autobiographical memories in groups of older adults, and adults with Alzheimer's disease, have a positivity effect as defined as “*…a relative preference in attention and memory for positive over negative information.*” The study further suggests that music cues in these groups evoke (positive) involuntary memories, strengthening the case for reminiscence music in a care setting. The apparent interconnectedness of emotion, memory, music, and our sense of self was further investigated by Baird and Thompson [35] in relation to Alzheimer's disease in old age. They suggest a framework that includes various aspects of self (ecological, interpersonal, extended, and private) and that “musical self-enhancement” as detailed by Elvers [36] can form part of a definition of self. Elvers advocated that musical experiences can be used as a tool to manipulate the affective state of the individual. This feature combined with the opportunities for social bonding and the reward mechanisms offered by music facilitates such self-enhancement. Baird and Thompson [35] suggested that this definition of self is something that can be preserved, to some extent, in people with dementia.

The combination of music, emotion, and autobiography is the core principle behind the longstanding success of BBC Radio 4's “Desert Island Discs.” As an archival resource, “Desert Island Discs” has been used by a study into career progression [37]; however, the potential for a systematic study related to reminiscence music is clear and one that is mirrored by the principles of the Memory Tracks application.

Reminiscence music, therefore, draws upon the emotional, narrative, and autobiographical connections made in life, particularly during formative years.

## 2. Materials and Methods

### 2.1. The Memory Tracks Mobile App

Memory Tracks is a care platform that utilises music associated to daily tasks. It is a technology platform that supports those people living with dementia, those caring for them, and their families. The application aims to help trigger memory, manage agitation, assist with care, and support daily routines through the benefit of song-task association. In its present form, Memory Tracks is an application accessed through an Android tablet device.

The Android operating system was selected as the platform for the development of the initial app. There were two reasons for this. Firstly, ease of development, the expertise available within the team, lower cost of future application programming, and flexibility with the operating system were most suited to using Android. Secondly, there was a consideration of the suitability and cost of tablet devices for the trial. The devices used were Lenovo Tab 3 730F and Lenovo Tab 7304F with front-facing speakers that offered suitable volume and clarity of the music. Rugged tablet cases and protective screens were added to ensure durability in the testing environment. With the objective to test Memory Tracks initially in care homes, the app was designed to be used offline, where WiFi Internet was not always available or desirable in this context. An additional benefit from this was that the tablets could be used in the airplane mode, thus extending the battery life of the device.

A total of 100 music tracks were preinstalled, which were pertinent to the age of the residents in the research trial. Memory Tracks established a partnership with Startle Inc., who manages the BBC Archives' Digital Music Juke Box that offered access to relevant music libraries of older recorded songs. With most of the residents in the study being 80 years and older, it required using songs primarily from the 1930s and 40s, as well as some from the 1920s and 50s. In order to identify the most memorable songs for residents, the research team accessed the online database (htttp://MusicVF.com) that listed the most popular 100 songs each year between 1900 and 2018.

For the Memory Tracks study, songs were played to groups within the study age range, and their level or recall was noted based on each individual's recognition of the music and lyrics. In the second phase of the research in Caernarfon, Wales, a number of the residents in the study spoke Welsh as their first language. A further 10 songs were added to the database that included well-known Welsh language songs and traditional folk songs.

**Figure 1:**
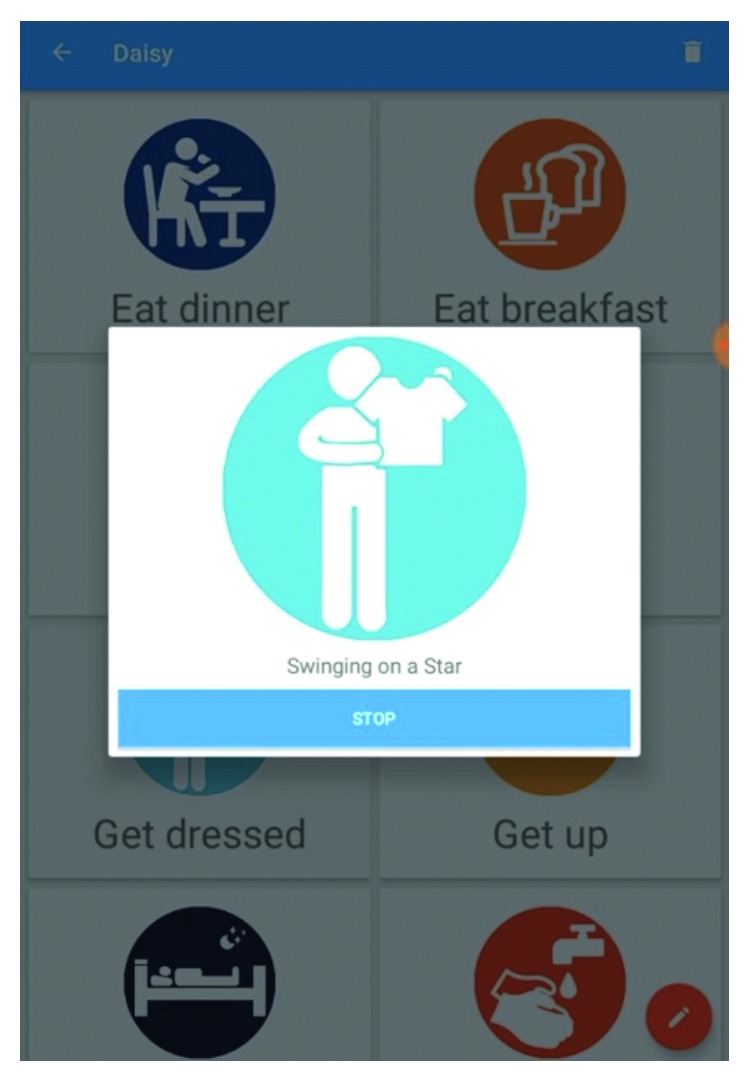
Screenshot of the mobile app with a task in progress (dressing).

The considerations for the application interface design were based on the principle user being a carer rather than the person living with dementia. With the initial research undertaken in care homes, it was necessary to acknowledge that professional carers would have a varied range of experience in using digital technologies. A further consideration was that using the app and completing the observation sheets would be an additional task in carers' daily work. Thus, clarity, simplicity, and speed were paramount in deploying the technology. The user interface of the app was structured around “tiles” associated to each song and task that would play the relevant music when tapped. During the early development process, these tiles used photographs to represent each task, with the objective that they would be personalised to each user. However, it became clear that the simplicity and speed of recognition for the app user needed to be improved. The tiles were subsequently changed to a standardised icon-based interface. Using the broad principles of Isotype (International System of Typographic Picture Education) [38], each task was assigned a pictorially representative icon (Figure 1).

**Figure 2:**
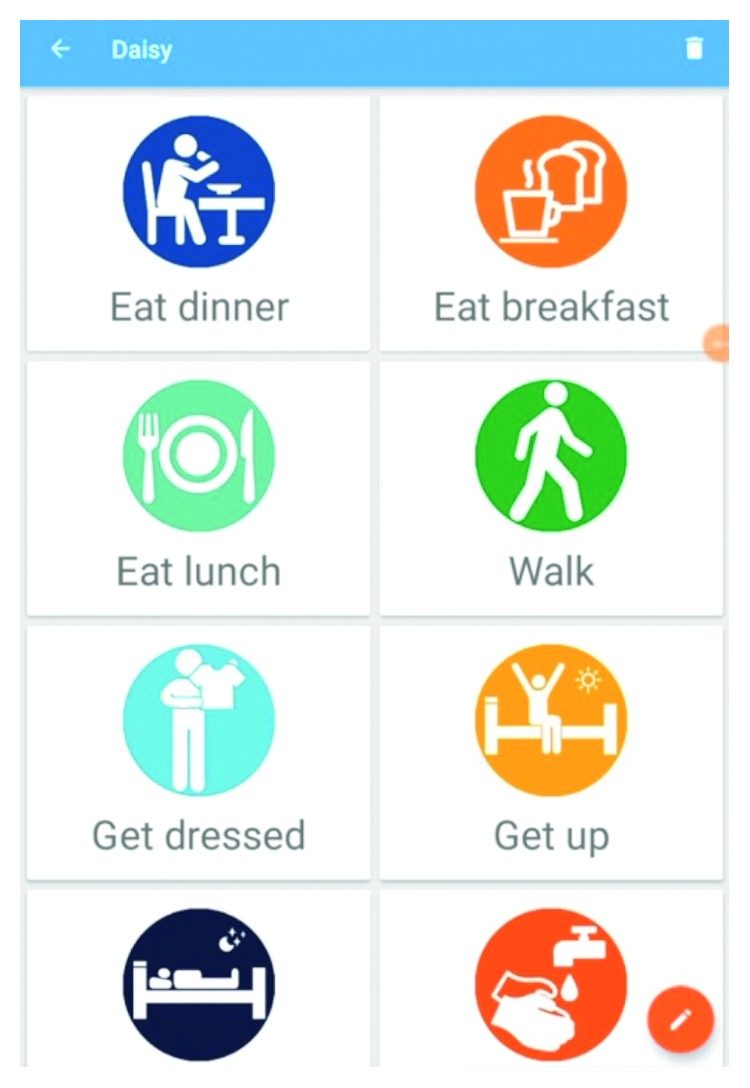
Screenshot of the mobile app showing a selection of the available tasks.

There was additional user recognition through the use of unique colours for each song-task icon. In addition to specific tasks, such as getting up, eating, or taking medication, generic alert icons were added for tasks unique to a particular person living with dementia, along with a function to add a text description to each tile (Figure 2).

During the development process, additional functions were identified and added. A radio icon was added that would play the songs stored in the app, but omitting any songs allocated to tasks. The research team was aware that music is frequently played in care homes, in both individual rooms and common areas. During interviews with the care home staff, it was apparent that this music was usually contemporary radio and of little relevance to the residents. Using the radio function would allow care home residents to experience reminiscence music that could be of greater relevance, potentially offering them therapeutic benefits.

## 3. Research Design and Methods

The research reported in this paper aimed to investigate the effect and impact of song-task association and musical reminiscence techniques, delivered via a mobile or tablet app, upon the wellbeing of a sample from the population of people living with dementia in UK residential care, as well as the care home staff supporting them.

It is anticipated that the association, and reduction in emotional behaviours, would be set in motion by the care home resident's first recognition of a song and the memory that it recalls for them. It could be assumed from the results of Cuddy and Duffin [39] that using music as the stimulus to build the association will provide a better recall of memory and association.

The present study was designed to facilitate the investigation of the mobile app in a small sample of care home residents with dementia at the Pendine Park Care Organisation homes from sites in Wrexham and Caernarfon, both in North Wales, UK. The research followed a cohort-based approach: establishing a set of baseline data in the initial phase, introducing the mobile app as an intervention, and then subsequently following up with each cohort. During the baseline period, the participating residents and care staff continued with their normal routine. Following this, the Memory Tracks app was introduced. Care staff were instructed to use the app with the residents selected for the study in conjunction with specific daily tasks, such as getting out of bed, taking medication, getting dressed, eating lunch, or receiving personal care. Before introducing the app to the care home, the researchers discussed each participating resident with members of the care staff to identify and prioritise a set of three or four key tasks that were challenging to accomplish. Relevant music was selected for each task from the database of songs available and matched to each resident using their demographic information: principally their year of birth and where they spent their childhood.

For each participant, we selected fourteen very well-known songs from when they were aged between three and twelve years, so as to cover a ten-year window, and associated each of these songs with a daily care task that they would be engaged with. The fourteen-song selection was unique for each resident. The most common tasks were dressing, going to the toilet, taking medicine, and bathing. These associated songs were played to the resident through the app whenever the care task was being performed. Some tasks were undertaken at fixed time slots, whilst others occurred at various points during the day. After each task, residents would generally perform other activities as part of their everyday life and were also encouraged to participate in the variety of daily enrichment activities available in the home. These enrichment activities include art, music workshops, quizzes, poetry, and reminiscence, or any activity of personal interest to each individual.

Music was played using the speakers built into the tablet provided to each of the participants. Headphones were not used so as to avoid complications associated with the additional equipment and isolation from their surroundings. It was recognised that when tasks were performed in communal areas or social settings, this may present an interaction effect with other residents at the home. However, an underlying intention in the study was to examine Memory Tracks in an ecologically valid scenario; hence, this was not controlled by the design of the research.

Care staff subsequently reported that, in communal areas, and particularly at meal times, they chose the music of the resident who was most likely to find that task difficult. This was done with the intention of providing a better environment for all concerned, with other residents often joining in. The music was played at a relatively low volume. In most cases, residents dined in small groups of two to four people or in their own room if that was their preference. If it was noted that the music bothered other residents, staff would relocate to a quiet area or a participant's room and continue to use the app there, but this was a very rare occurrence as most other residents enjoyed and responded positively to having the music playing, mainly by singing along or humming to the track.

Two cohorts of participants from each site took part in the study. Prior to the app being introduced, the researchers visited the care home to train the care team in the use of the Memory Tracks software platform. In Wrexham, the residents were observed for approximately three weeks before the mobile app was introduced and then for four weeks whilst using the mobile app. In Caernarfon, the same process was repeated for two weeks and four weeks, respectively. During the period where the app was being used, care staff were asked to play the relevant songs when the associated care tasks were being performed. Quantitative data were collected over the course of the study period, and qualitative data were captured at the end of the study period.

In terms of quantitative, observational data were collected daily by the care staff using a paper-based form and with a small number of questions. The care staff received a training session about the observation form prior to the start of the study. A deliberate design decision was taken to make the form a simple and fast process to complete, so as not overly to distract the staff from their usual duties. Six measures were taken each day. Three of these measurements used the Self-Assessment Manikin (SAM) pictorial scale [40], using ordinal intervals between 1 and 9, to capture each resident's emotion on the dimensions of *valence* (happy to sad), *arousal* (excited to calm), and *dominance* (dependent to independent). The other three measures were questions taken from the Quality of Life in Alzheimer's Disease (QOL-AD) questionnaire [41], measured using ratings of “poor,” “fair,” “good,” and “excellent,” to assess each resident's *physical health*, *memory*, and *life as a whole*. One set of measures was recorded for each participant each day during the study. The observations were scheduled so as to gain a balance of measurements taken at different times during the day (morning, afternoon, and evening).

Following the end of each study period, qualitative data were obtained by conducting a set of semistructured interviews with the care staff. The prompts used during the interview were designed to elicit responses from care staff on several topics: setting up the app with the participant, the app's ease of use, impact upon the care staff's own work, and perceived impact of the app upon the residents, along with any additional comments or issues. Interviews were conducted on the care home site and had a mean duration of 14 minutes and 48 seconds. There were always two or more members of the research team present. Some interviews were with small groups of care staff, and the others were with individuals. After the data were collected, the interview recordings were transcribed by members of the research team, then double and triple checked, and subsequently analysed using thematic analysis [42], which resulted in main themes and subthemes being formed from recurring statements or sentiments in the study.

## 4. Participants

The selection of participants was undertaken with the advice of the staff at Pendine Park Care Organisation. In doing so, participants could be identified based upon their current physical and cognitive characteristics, including the level of dementia with which they are living, resulting in a convenience sample. Participants were residents recruited from two care homes in the Pendine Park group. Participants were living with dementia, were typically between levels 5 and 6 on the Global Deterioration Scale for Assessment of Primary Degenerative Dementia [43], and were included in the study to reflect different types and stages of dementia. Each individual was selected by the registered manager and care team at each site. The balance of the individuals participating in the study cohort was those with Alzheimer's disease (60%) and vascular dementia (40%). The care team selected a mix of residents, some of whom were recognised as enjoying music and others that had difficulties engaging with their routine tasks, such as bathing and eating. Informed consent was obtained either from each participant directly or via an appropriate designated person [44]. Where the legal responsibility of care lays with a family member or guardian, a discussion was held, and they were provided with formal information about the study before consent was requested. In other participants, where the care home management held this responsibility, the same process was followed. In order to safeguard participants, care staff were instructed to take the intervention away if it caused distress or negative behaviours.

In the Wrexham component of the study, a total of six residents were recruited. For the portion of the study based at the Caernarfon site, a total of eight residents were recruited, although only four of these fully completed the quantitative part of the study. Overall, there were 5 male and 9 female participants with a mean age of 84.60 (SD = 8.69) years, with an overall age range of 69 to 97 years. Participants were mainly living with diagnoses of vascular dementia or Alzheimer's disease and had a mixture of idiosyncrasies and conditions. In many cases, these could be problems with compliance in performing everyday tasks, such as washing, eating, and dressing, or in communication. In some cases, this could relate to being physically vulnerable and at risk of falling or could include mood swings and aggressive behaviours.

## 5. Results and Discussion

### 5.1. Quantitative Results

Data were collected at the two sites for the periods described earlier in Methods and Materials. Upon receipt of the paper-based observation sheets and their transcription to digital format, it became apparent that there were occasional inconsistencies and missing data, particularly at the Caernarfon site where there were only usable data collected for four of the eight participants. This was further compounded by the duration of data collection being slightly different at each site. Consequently, given the relatively small number of participants (*n* = 10) and rather than applying a method to interpolate or impute missing values, the decision was made to use mean values for each participant, treating them as a single cohort, before and during their use of the mobile app, for each of the six quantitative measures. Since the main aim was to investigate any difference within subjects over the course of the research, all of the data collected from the participants were able to be utilised, albeit in a descriptive form.

To investigate any effect of the mobile app intervention upon the participants, a one-way MANOVA with repeated measures was performed upon participants' mean values for the six measures. The statistical test showed no significant effect (Wilks' lambda *F*(6, 4) = 0.29, *p*=0.33), indicating that the use of the mobile app had neither a positive nor a negative impact upon the six combined indicators of participants' wellbeing. Although it was not a normal process following the MANOVA outcome, given the small number of participants and combination of indicators, univariate analysis for each of the six measures was performed. Two of the measurements showed significant results: *valence* (*F*(1, 9) = 7.232, *p* < 0.05) and *physical health* (*F*(1, 9) = 6.000, *p* < 0.05). Whilst these findings must be viewed with caution, they provide an initial indication that the use of the mobile app may have positive benefits for users.

**Figure 3:**
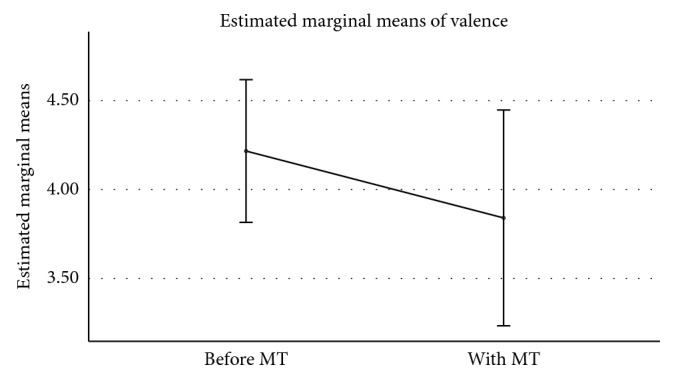
Valence ratings before and during use of the mobile app (Memory Tracks). Error bars: 95% CI.

As shown in Figure 3, participants' scores for emotional valence decreased, on average, from 4.22 to 3.84. Recalling that the scale for valence operated on a value of 1 being labelled *happy* and 9 labelled *sad*, this movement is towards the positive valence end of the scale.

**Figure 4:**
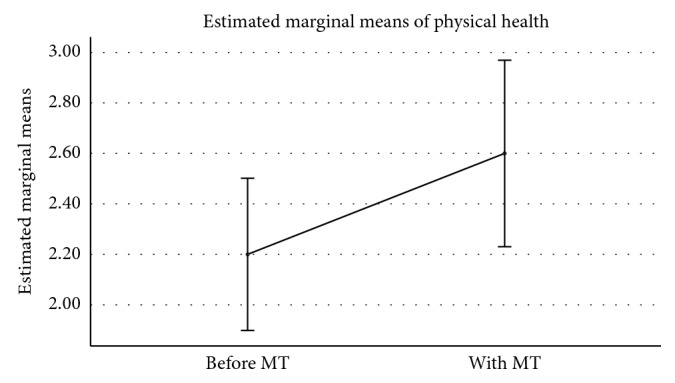
Physical health before and during use of the mobile app (Memory Tracks). Error bars: 95% CI.

Figure 4 shows participants' scores for the ratings of physical health increased with the use of the mobile app, on average, from 2.20 to 2.60. Recalling that the scale for physical health operated on a value of 1 being labelled *poor*, 2 labelled *fair*, 3 labelled *good*, and 4 labelled *excellent*, this movement is towards the positive physical health end of the scale.

Given the findings from the inferential analysis, and cognisant of the number of participants, there is value in analysing the data on a descriptive level. For the questions taken from the QOL-AD relating to factors of physical health, memory, and life as a whole, mode responses are referred to. These were obtained by calculating the mode of all of the observations, per participant, before Memory Tracks was introduced and again for the period where Memory Tracks was present. By examining the individual participants, we can make the following observations:

- There was no definitive change on the SAM *arousal* scale (5/10 moved towards “Calm,” and 5/10 moved towards “Excited”).
- 8/10 residents showed small changes in terms of *dominance* (moving towards the “Independent” end of the scale).
- The mode response in 4/10 residents increased one position from “Fair” to “Good” for the QOL-AD question “How would you rate the resident's physical health today?” In the remaining six residents, the mode of their scores remained the same.
- The mode response in 2/10 residents increased one position from the ratings received prior to Memory Tracks being introduced for the QOL-AD question “How would you rate the resident's memory today?” One moved from “Poor” to “Fair” and the second from “Fair” to “Good.” In the remaining eight residents, the mode of their scores remained the same.
- The mode response in 3/10 residents increased one position from “Fair” to “Good” on the scale for the QOL-AD question “How would you rate the resident's *life as a whole* today?” In the remaining seven residents, the mode of their scores remained the same.

### 5.2. Qualitative Results

Themes were found through the quotes and reoccurring benefits and limitations highlighted about the mobile app that were repeated in each of the interviews. The themes identified are *behaviour*, *ability*, *impact*, *routine*, and *music*, with subthemes providing some additional clarity and definition to the main themes.

#### 5.2.1. Theme: Behaviour

Music can influence mood and behaviour in a significant way. Care staff who took part in the interviews believed that the behaviours changed due to the mood of the individual shifting. This could be seen in some of the examples that were given by the care staff such as

- “*…he would have known what the music was for but it did calm him down really*”
- “*Quite nice for us not to have her screaming*”

Sundown syndrome [45] was mentioned a number of times by interviewees. This is when a resident's mood becomes negative due to the time of the day. However, music whether it was being played in the lounge or via the app seemed to help this mood from becoming too dark, especially at bedtime.

#### 5.2.2. Theme: Ability

Reduction in physical and cognitive abilities is often seen in people living with dementia, especially when compounded by the natural ageing process. The presence of music via the app was perceived as encouraging some participants to unlock abilities that had deteriorated. For example, one participant was able to feed herself once the app was available at meal times. The theme reoccurred in a number of interviews with care staff:

- “*I know* [participant name] *loves it*, *downstairs*, *she likes the memory of it*”
- “*When she tells me she needs the toilet she grabs the tablet as if to tell me ‘let's go*”

Whilst there were improvements in physical and cognitive abilities for some of the participants, a third element of ability appeared to develop, that of independence. It appeared that the app allowed some participants to do small tasks independently that they had not been able to before, thereby improving their motor skills and memory of doing it prior to the onset of their condition:

- “*Yeah*, *she independently eats her meals and drinks*”

#### 5.2.3. Theme: Impact

In the data where this theme reoccurred a number of times, it appeared that there was only an impact on some of the residents. These participants tended to be those who had a previous interest in music, or had worked with, or played music when they were a younger:

- “[participant name] *went back to her agitated mood because we didn't have the tablet there*”
- “*It was beneficial for* [participant name] *but not as much for* [another participant name]”

From the data, it appeared that any impact that the app had was positive, with the exception of one resident. This participant in the study recognised a song that had an unpleasant memory for her. In this instance, the song was removed. Although it was a negative experience for the participant in question, this finding shows another form of impact that music as a stimulus for recall can have and supported the requirement for new associations to be created in place of older ones.

#### 5.2.4. Theme: Routine

Routine was an expected theme to develop, and there were questions in the interviews that sought to determine its presence. The app was created to focus on using music through the care routine of a resident. There were two facets to the theme of routine—one was the way that the app fitted into the care staff's daily routines and the other was the way playing music from the device influenced the resident's routine:

- “[participant name] *loves it*, *when she is having breakfast and dinner*”
- “*I would actually make time*, *it doesn't matter how busy I am I would always make time to actually use this (the app) with them*”
- “*…with* [participant name] *it relaxed him and he was calm*, *you get the tasks done quicker*”

#### 5.2.5. Theme: Music

Music is one of the key components and concepts in this research; therefore, it was strongly anticipated that it would be an overall theme resulting from the qualitative data. There were a variety of ways in which the music theme occurred in the data—from the care staff learning the words of the songs and singing along with the residents to the type of music that the residents would respond to:

- “*I think the type of music would have helped too as* [participant name] *like reggae music*”
- “*He said a couple of times ‘Oh I knew this song when I was a boy*”
- “[participant name] *absolutely loves music*”

As established literature has shown, music therapy has demonstrated significant improvement for health and wellbeing over and over again. Although the results of the present study show once again that music can impact the wellbeing of participants, it has also produced new themes and quantitative data that suggest music can be used as a daily aid in care, not just as a therapy or for social activity. More research with a larger sample would be needed with further research to support fully the benefits of the Memory Tracks app as a daily aid in music task association. From the research presented here, it can be seen that some residents are starting to build a foundation association with the app and music and slowly associating that music with their daily tasks. However, for such a specific association to be made, it may take a longer time than the duration of our study.

## 6. Conclusions

Studies in music and memory have shown how there may be a reserve of memory specifically for music, and this may be why music can trigger autobiographical connections or associations in memory despite a cognitive impairment. It was the aim of this research project to consider the impact of music not only on building association but also on the resident's wellbeing. This was clear to see when care staff explained how certain residents were not as agitated or were participating in sessions and tasks where they had not previously done so.

The quantitative results here are best considered in partnership with the qualitative analysis, to form a more holistic view of the impact that Memory Tracks had upon the residents of Pendine Park. The individual nature of each participant, their background and demographic, may well impact their amenability to using reminiscence music, and this may account for the indications that the mobile app has a small, positive effect upon some residents but not on others. The quantitative results provide early indications that the mobile app may have a beneficial impact, but a larger study is required to provide a better picture, and this is a priority for future research using the app, although recruitment of a large cohort and maintaining control over the data collection routines are shown to be problematic in such a study, though not insurmountable.

This research has shown that residents may make a general association between the Memory Tracks app and the music that is played. This would be a general association that shows a positive impact and indication that a specific connection between a song and a task could be made. El Haj et al.'s research [46] on the association and memory supports this process and also highlights why music has such a strong stimulus. It identifies that people regularly tie their emotions to music and, as a by-product of this, to memories.

More generally, extensions of the present work, if successful, may produce benefits in people living with dementia across the full spectrum of the condition, from those with primary care needs to those still living independently. In the future, we anticipate that the work will be extended to see if benefits can be translated to other neurological conditions, such as stress, depression, and autistic spectrum disorder.

One avenue for future work would be to focus upon the specific effects of music therapy in people living with dementia through the use of neurophysiology techniques. Such an approach is attractive since it would give an alternate and objective way to measure the effects of music and song-task association, as well as the ability to observe these effects in real time. An example of such an approach exists in the work of Mitterschiffthaler et al. [47], who used functional-magnetic resonance imaging (f-MRI) to investigate positive and negative affective states in response to listening to Western classical music. Similarly, Rojo et al. [48] used f-MIR and transcranial magnetic stimulation (TMS) to evidence that a programme of music-supported therapy was a “…useful neurorehabilitation tool in patients with chronic stroke and leads to neural reorganization in the sensorimotor cortex.” Both of these articles lend credence to the suggestion that music therapy induced demonstrative brain responses and that these may have neurotherapeutic benefits.

There are further technology upgrades planned to the Memory Tracks app. A greater understanding of the application after the initial study has indicated the following developments:

- Improvements in the music selection process: the aim is for the app to identify possible reminiscence tracks automatically, based on the birth date and location of the early years of the subject living with dementia. As memorable music is identified for each subject, a recommendation engine will suggest further songs.
- Improved survey data collection: in future iterations, the observation sheet will be included in the app, with a daily prompt to complete the information. This will reduce the additional tasks for carers and support easier analysis for larger sample groups.

Further technology developments are planned as part of the Memory Tracks enterprise. These include the addition of the sensor technology to add contextual and behavioural triggers. Additionally, the use of machine learning will be implemented to predict typical behaviours and send alerts to carers where behaviours are abnormal.

## Data Availability

The data used to support the findings of this study are available from the corresponding author upon request.
